# Comparison of nifekalant and amiodarone for resuscitation after cardiopulmonary arrest due to shock-resistant ventricular fibrillation

**DOI:** 10.1186/cc9711

**Published:** 2011-03-11

**Authors:** N Harayama, S Nihei, Y Isa, H Arai, T Shinjou, K Nagata, M Ueki, K Aibara, M Kamochi

**Affiliations:** 1 University Hospital of Occupational and Environmental Health, Kitakyushu City, Japan

## Introduction

Nifekalant (NIF) is a pure potassium channel blocker developed in Japan and it has been used widely for treating fatal ventricular tachyarrhythmia since 1999. Because intravenous amiodarone (AMD) was approved in 2007 in Japan, there have been few studies about the comparison of the efficacy of NIF and AMD for resuscitation after cardiopulmonary arrest patients due to shock-resistant ventricular fibrillation.

## Methods

We performed a retrospective study in 32 consecutive cardiopulmonary arrest patients treated by NIF or AMD due to more than twice shock-resistant ventricular fibrillation from April 2005 to October 2010. The statistical analyses performed by chi-square test and nonpaired *t *test.

## Results

The mean (± SD) age was 62.2 ± 16.1 years and 25 of 32 were male patients. All 32 patients were treated with tracheal intubation and intravenous epinephrine. Seventeen patients received NIF administration and 15 patients received AMD. The average initial administration dose of NIF was 11.1 ± 3.4 mg and that of AMD was 171.7 ± 59.7 mg. The rate of return of spontaneous circulation (ROSC) was 41.2% (7/17) in the NIF administration group and 26.7% (4/15) in the AMD group. The survived discharge rate from our hospital was 29.4% (5/17) in the NIF group and 13.3% (2/15) in the AMD group. There were no significant differences between the two groups with the rate of ROSC and survived discharge. The mean interval from the antiarrhythmic drug (NIF or AMD) administration to ROSC was 7.8 ± 6.6 minutes (NIF) and 19.9 ± 11.7 minutes (AMD). There was significant difference between the interval of NIF and that of AMD (*P *< 0.05).

## Conclusions

Although NIF is an anti-arrhythmic agent for life-threatening ventricular tachyarrhythmia, it does not have negative inotropic activity. NIF changes shock-resistant ventricular fibrillation to spontaneous circulation more quickly than AMD. NIF is strongly effective for resuscitation of shock-resistant ventricular fibrillation.
